# Influences of the Built Environment on Rural School Children’s Travel Mode Choice: The Case of Chengdu

**DOI:** 10.3390/ijerph19159008

**Published:** 2022-07-25

**Authors:** Haimei Li, Li Han, Yibin Ao, Yan Wang, Tong Wang

**Affiliations:** 1 Humanities and Law School, Chengdu University of Technology, Chengdu 610059, China; lihaimei2012@cdut.edu.cn; 2 College of Environment and Civil Engineering, Chengdu University of Technology, Chengdu 610059, China; hanli0713@163.com; 3 Department of Engineering Management, Sichuan College of Architectural Technology, Deyang 618014, China; wangyan@scac.edu.cn; 4 Faculty of Architecture and Built Environment, Delft University of Technology, 2600 GA Delft, The Netherlands

**Keywords:** urbanization, rural built environment, school travel, the multinomial logit model

## Abstract

Since the reform and opening up of China, the rural built environment has changed dramatically. There is a need to understand how such changes have impacted rural children’s school travel mode choice to design the built environment and plan schools accordingly. This paper combines field measurement methods and questionnaires to obtain data on rural children’s school travel behavior and uses the multinomial logit (MNL) model to investigate the impacting factors. The results show the following insights: Age has a significant positive impact on children’s choice of bicycles and buses. The improvements in road layout and facility conditions are significantly and positively associated with children’s choice of electric bicycles for school. There is a significant positive correlation between a good and safe public environment and children’s choice of cycling. Furthermore, distance from home to school has a significant impact on the choice of children’s school travel mode: the greater the distance to school, the higher the probability that children will choose motorized modes of travel such as buses and private cars. This study provides empirical data and evidence in designing rural transport systems for school children based on their preferences concerning built environment factors.

## 1. Introduction

**Figure 1:**
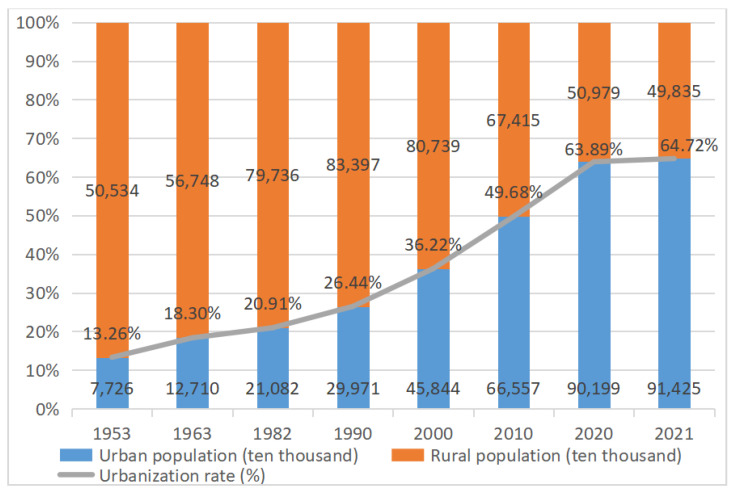
Urbanization rate in China.

Urbanization refers to the historical process of gradual transformation of a county or region’s society from a traditional rural-type society dominated by agriculture to a modern urban-type society dominated by non-agricultural industries such as industry and services as a result of the development of social productivity, scientific and technological progress, and industrial restructuring [1]. Since the reform and opening-up, China’s rural urbanization process has entered an accelerated state, with a rural population of 509.79 million, accounting for 36.11% of the country’s total population, and an urbanization rate of 63.89% according to the 2020 census data in China Statistical Yearbook 2021 [2,3,4] (see Figure 1). From the figure, it can be seen that China’s urbanization rate reached 63.89% in 2020 and is expected to reach 65% by 2030. The rapid urbanization of rural areas has also caused a series of problems, such as the increased pressure on arable land, overcrowded urban population, inconvenient transportation, etc. The original housing structure and transportation structure of rural residents are also changing along with the urbanization process.

The relatively large Chinese rural school-age children group generates a large travel demand. However, the number of rural schools shows a trend of reduction as these schools become more concentrated. At the beginning of the 21st century, China’s Ministry of Education launched the school mapping restructure (SMR) plan, which requested closing small rural schools, opening larger centralized schools in towns and counties, and shifting from “running schools in villages” to integrating the resources of nearby schools. In this process of merging, elementary schools in poor mountainous areas are the most heavily and profoundly affected, resulting in greater distance to schools for many rural students and a small number of urban students. Many children have lost the opportunity to study in their rural schools [5]. The school mapping restructure plan has brought great inconvenience to rural children and parents. As a result, rural school-age children are choosing boarding schools, which changes the way they go to school.

For available travelling modes, in recent years, with the development of the economy and the acceleration of urbanization, car ownership has increased rapidly. China has become a car society nationally. Data from the Ministry of Public Security show that by the end of March 2022, the number of motor vehicles in China reached 402 million, including 307 million cars. The number of motor vehicle drivers reached 487 million, including 450 million cars [6].

Furthermore, the development of rural road hardening projects, the vigorous development of road network construction, and the improvement of rural residents’ living standards have also led to an increasing number of electric bicycles owned by rural residents and a decrease in bicycle ownership year by year. Moreover, the low building density has also led to an increase in the use of cars by rural residents [7]. The increase in rural car and electric bicycle ownership not only changes the travel structure of rural residents but also raises problems such as travel safety and traffic congestion.

The World Health Organization performed a survey and noted that traffic accidents are the number one cause of child deaths worldwide every year, and this is especially severe in China. Globally, approximately 5 million people are killed in road traffic accidents each year. More than 70% of them occur in developing countries. Moreover, 80% of these accidents are related to children. This is a serious social problem. According to the statistics of China’s transportation department, more than 18,500 children die in traffic accidents in China every year. This mortality rate is 2.5 times that of European countries and 2.6 times that of the United States [8]. In recent years, school-age children’s traffic accidents occur frequently. It is very important to create a safe and secure travel environment for school-age children while going to and coming from school. We should not only pay attention to the rights and interests of urban children but also pay attention to the rights and interests of rural children.

## 2. Context and Literature Review

Liu believes that the physical environment can influence a child’s choice of travel mode to school, and the physical environment includes the natural physical environment and the built environment [9]. This study focuses on rural school-age children and mainly studies the impact of the built environment on children’s school travel mode choice. Therefore, the research scope of the built environment is reduced to rural school travel built environment.

School travel is an essential activity in the daily life of school-age children, which mainly refers to the regular round-trip activities between school and residence. At present, the school travel modes of school-age children in China mainly include walking, cycling, taking public transportation, using an electric bicycle, being driven in a private car, using school buses, etc.

Since 1980, scholars have shifted their focus from adult travel to children’s school travel. Early research on public health linked childhood obesity to travel safety, and walking and cycling to school are considered active travel modes which can reduce obesity. Furthermore, they early research shows that students’ active commuting to and from school (e.g., walking, bicycling) has many social benefits [10,11].

For the built environment factors impacting children’s travelling mode choice, LaRouche et al. concluded that school traffic intervention may improve students’ school travel mode choice [12]. Medeiros et al. noted that “school travel planning” may help to change the mode of school traffic and facilitate students’ active traffic [13]. Individual and family characteristics, parents’ characteristics, and attitudes impact children’s decision to actively go to school as well [14,15,16].

The school duration and population density around schools have a significant impact on the choice of travel mode [17,18,19]. Müller et al. found that partial school closures (similar to China’s “withdrawal and merging” policy) have a negative impact on students’ school travel mode, and students may shift from a low-cost transportation mode to a high-cost transportation mode [20]. Distance, traffic speed, traffic volume, intersection safety, and weather conditions were concluded in the study as the top five barriers to active travel for children [21]. In addition to this, Dalton et al. found that the frequency of active commuting is correlated with the season for rural children [22].

A built environment with high shade tree density and paved sidewalk integrity encourages children to walk to school independently [23,24]. The increase in the number of street areas and intersections hinders children from going to school on foot independently. Safe neighborhood environments increase the possibility for students to walk to and from school independently [25]. Children’s active school travel is positively correlated with children’s autonomous mobility, children’s perception of neighborhood safety, parents’ perception of social interaction, and the neighborhood social environment [14]. The built environment plays an important role in children’s school travel and feeling of safety, which even goes beyond the closely related variables such as the social, cultural, and economic status of the community and its members. There are many studies on the impact of the built environment on travel mode. In the research on the impact of the built environment perception on school travel, there are scholars who have measured children’s perception of the school environment through questionnaires to explore the factors that influence children’s school activities [26]. Huertas Delgado et al. found that parents’ perception of distance and intersection safety influence children’s school travel mode choices in Ecuadorian children [27]. In the study on the impact of the built environment perception of Greek teenagers on general learning, it was found that poor lighting and lack of a sidewalk-built environment limits rural teenagers’ choice to walk to school [28]. 

The research on children’s school travel behavior started late in China, and the research results are relatively limited. Similar to existing studies, the factors influencing school-age children’s travel mode choice in China include individual child factors, family and household member factors, transportation factors, and built environment factors. The choice of travel mode of school-age children can be affected by gender, age, or both [29,30]. Among household and family member factors, household income, household transportation ownership, whether someone is accompanied, car ownership, and parental commuting attributes all affect parental pick-up and drop-off decisions and children’s choice of travel mode [31]. Using Hangzhou City as an example, Tang found that students’ satisfaction with the traffic conditions in the neighborhood and the built environment have significant effects on secondary school students’ choice of travel mode to/from school [32]. The characteristics of transportation facilities, mixed land use, and population density around the school are positively correlated with the possibility of students walking to school and negatively associated with the probability of going to school by non-active transportation modes such as bicycles, electric bicycles, or cars [33]. School-age children’s cognition of traffic safety, commuting distance, road traffic safety, and parents’ commuting attributes affect parents’ pick-up and drop-off decisions [34]. In addition, Xue et al.found that air quality may have an impact on children’s active travel mode [35]. Shen et al. studied the influencing factors of three types of built space environment, namely residential environment, school environment, and urban environment on children’s independent travel ability [36]. Through the empirical investigation of children’s independent travel ability in two primary schools in the center of Yueyang, this paper explores the planning countermeasures to improve children’s autonomous travel ability suitable for China’s conditions and provides empirical support for creating a child-friendly built environment. Furthermore, it was found that due to the long distance to school, the proportion of bicycles among elementary school students traveling to school in Beijing decreased from 45.8% in 2000 to 20.1% in 2006, while the proportion of cars increased eight times [37]. In Nanjing, electric bicycles have become a new trend for elementary school students to travel to school [38].

Besides the empirical studies mentioned above, Ikeda et al. performed a literature review for English articles published between January 2000 and July 2017 and concluded that positive school travel is positively correlated with safety, walking, and neighborhood social interactions, and negatively correlated with travel distance and car ownership [39]. Wang et al. summarized results of research for the built environment impacts on school travel, and categorized the influencing factor systems of the built environment into four aspects: land, transportation, design, and school [40]. 

By reviewing the literature on the influencing factors of school-age children’s travel to school, it was found that in recent years, domestic and foreign scholars have explored the influencing factors of students’ travel mode choices in different countries, regions, and in different age groups. These research provide a good foundation to conduct empirical research for rural areas in China, but further empirical research is needed as the indicators and attributes identified previously might not be completely consistent with the rural China conditions and the rural children’s preference. 

Furthermore, despite the rapid development of rural areas in recent years, there is a large gap between urban and rural areas in terms of built environments, school layout, and road conditions. The conclusions of the study on the impact of the urban built environment on children’s school travel behavior cannot be fully applied to rural areas. At present, the relevant research by scholars on children’s school travel in rural China and their influencing factors are in the exploratory and initial stage, which limits the further planning of new countryside and urbanization. This study aims to use empirical data collection and analysis to help design a more suitable built environment for children which promotes active travelling modes. This study also aims to provide some reference for rural infrastructure planning, construction, and optimization to create child-friendly rural areas. Since children’s perceptions of their built environment influence children’s school travel mode choice, this study used the variable of rural children’s perceptions of the school travel built environment to explore the influence of the subjective built environment on children’s school travel mode choice.

## 3. Methodology

This section firstly specifies the models to be used (Section 3.1) and then explains the data collection process (Section 3.2) and the variables applied (Section 3.3). The multi-collinearity of variables test was performed and is shown in this section as well (Section 3.4). 

### 3.1. Model Specification

#### 3.1.1. Factor Analysis

Factor analysis refers to the statistical technology of extracting common factors from variable groups. The principle is dimensionality reduction. Starting from the study of the dependence within the original variable correlation matrix, some variables with complex relationships are reduced to a few comprehensive factors. The theoretical model of factor analysis is generally expressed as follows:

Suppose M samples and P indicators are available; $X:{\left(X_{1},X_{2},\ldots \ldots \;,X_{P}\right)}^{T}$ is a random vector. The common factor that should be determined is $F:{\left(F_{1},F_{2},\ldots \ldots \;,F_{m}\right)}^{T}$

(1) $$X_{1}=a_{11}F_{1}+a_{12}F_{2}+\ldots +a_{1m}F_{m}+\varepsilon_{1}$$

(2) $$X_{2}=a_{21}F_{1}+a_{22}F_{2}+\ldots +a_{2m}F_{m}+\varepsilon_{2}$$

(3) $$X_{p}=a_{p1}F_{1}+a_{p2}F_{2}+\cdots +a_{pm}F_{m}+\varepsilon_{p}$$

The previous equations compose the factor model. Matrix $A=\left(a_{ij}\right)$ is the factor load matrix and $a_{ij}$ is the factor loading. A is the correlation coefficient between a common factor $F_{i}$ and variable $X_{i}$. ε is a special factor that represents factors other than the common factor [41].

#### 3.1.2. Multinomial Logit Model

The existing research of studying built environment impacts on students’ choice of school travel modes mainly establish the Discrete Choice Model, which is suitable for the analysis of the behavior choice of many school travel modes such as walking, cycling, and public transportation [42,43]. The multinomial logit model (MNL) and multinomial probit model (MNP) have certain advantages to fit travel mode choice. The main differences between the two models are: MNL specifies that the residual items are drawing from independent extreme value distributions, while MNP assumes that the residual items are normally distributed with standard deviations. In this study, we used the research data to fit these two models and the model results are generally consistent, but the 2 Log Likelihood value of MNL (988.596) is smaller than that of MNP (993.635), which means that the data fit MNL better. Finally, MNL is selected for further analysis and interpretation.

Assuming that the effect of the nth investigated child choosing the ith school travel mode is $U_{\mathrm{ni}}$ and $J_{n}$ is the scheme set, then, $i\in J_{n}$, $U_{\mathrm{ni}}=V_{\mathrm{ni}}+\varepsilon_{\mathrm{ni}}$, and $V_{\mathrm{ni}}=\beta^{\prime }X_{\mathrm{nk}}$. Among them, $\varepsilon_{\mathrm{ni}}$ is the random error term; $X_{\mathrm{nk}}$ is the nth factor affecting the *n*th child’s choice behavior; $\beta^{\prime }$ is the parameter to be estimated. Then the probability of the nth investigated child choosing the ith travel mode is:

(4) $$\begin{array}{c} P_{n}\left(i\right)=\mathrm{Prob}\left(U_{\mathrm{ni}}\geq U_{\mathrm{nj}},j\in J_{n},i\neq j\right)=\mathrm{Prob}\left(V_{\mathrm{ni}}+\varepsilon_{\mathrm{ni}},j\in J_{n},i\neq j\right)\\ =\mathrm{Prob}\left[V_{\mathrm{ni}}+\varepsilon_{\mathrm{ni}}\geq \underset{j\in J_{n}}{\max\left(V_{\mathrm{nj}}+\varepsilon_{\mathrm{nj}}\right)}\right] \end{array}$$

If each random item is subject to independent identically distributed, then:

(5) $$f\left(\varepsilon_{1},\varepsilon_{2},\ldots ,\varepsilon_{n}\right)=\prod_{n}g\left(\varepsilon_{n}\right)$$

$g\left(\varepsilon_{n}\right)$ is the distribution function corresponding to the nth investigated child. Assuming that $g\left(\varepsilon_{n}\right)$ obeys a dual exponential distribution, then the probability of selecting the ith mode of school travel Jn is as follows:

(6) $$p_{\mathrm{in}}=\frac{\exp\left(V_{\mathrm{in}}\right)}{\Sigma_{j\in J_{n}}\exp\left(V_{\mathrm{jn}}\right)}=\frac{1}{\Sigma_{j\in J_{n}}\exp\left(V_{\mathrm{jn}}\right)-\Sigma_{j\in J_{n}}\exp\left(V_{\mathrm{in}}\right)}=\frac{\exp\left(\beta^{\prime }X_{\mathrm{nk}}\right)}{\Sigma_{j\in J_{n}}\exp\left(\beta^{\prime }X_{\mathrm{nk}}\right)}$$

### 3.2. Data and Sample Collection

Even though Sichuan’s rural areas are huge, there is little difference in the spatial form of the same village, according to our previous studies and site visits (86 villages were visited). In this study, the researchers tried to select as many sample villages as possible to more broadly represent the reality of rural areas in Chengdu, Sichuan. For each sample village, as long as there are several valid sample villagers (households), the overall situation of the whole village can be represented. The most difficult aspect of rural research is to obtain effective research data because detailed statistics and internet data are not available for rural areas. To obtain effective research data, the research team carried out an in-depth village-to-household survey. Although this approach has certain limitations, it is a very effective way to obtain research data in rural areas.

This research takes rural children in Chengdu, Sichuan Province as the research objects, and the data were obtained from a survey on the school travel mode of rural children conducted by the Department of Construction Management of the Chengdu University of Technology in July 2021. The initial selection of sample areas and sample villages was carried out by collecting information online. The candidate villages were selected based on their accessibility to schools and transportation networks status. Before the formal research, the research team conducted a pre-study and screened the candidate villages according to their vigilant psychology and cooperation. As a result, eight areas were selected in this study, including Wenjiang District, Xindu District, Longquanyi District, Pidu District, Chongzhou City, Qingbaijiang District, Jianyang City, and Pengzhou City, involving a total of 33 villages. Researchers learned about the situation of each village by communicating with students and using maps. Villages that were easily accessible to researchers, well-developed, and contained schools or had schools nearby were selected, and several nearby villages were alternatively selected to prevent situations such as insufficient sample size in the chosen villages.

Researchers conducted random visits and face-to-face questionnaire surveys with rural children and guardians to complete the data collection of personal school travel conditions, family, and socio-economic conditions, and built environment perception. The investigators used the Ovi map mobile phone software to determine the location of the site, measure the distance between the residence of rural children and their schools, and obtain data on the built environment.

### 3.3. Variable Description

Through the researcher’s household research in 33 villages, the number of valid questionnaires collected was 638. In this study, the dependent variable travel modes are defined as six categories: walking, bicycle, bus, private car, electric bicycle, and motorcycle. Among them, the largest proportion, 58.60%, chose electric bicycles to go to school; followed by private cars, walking, and buses, 14.10%, 13.20%, and 9.20% respectively; the proportion choosing bicycles and motorcycles for school travel was relatively less, which were 3.40% and 1.40% respectively. School travel built environment, travel distance/time, and sociodemographic variables were considered to be the key factors affecting the choice of travel mode in much literature. At the same time, the perception of school travel built environment also affects the choice of travel mode for rural school-age children to school.

#### 3.3.1. Sociodemographic Variables

The sociodemographic variables used in this chapter of the study include Children’s gender, age, grade, and whether they are the only child. To supplement the understanding of children’s family situations, a sociodemographic survey was conducted with the guardians of the children interviewed. The variables include gender, age, total personal and family income, education level, whether they hold a driver’s license, and the number of family private cars, motorcycles, electric bicycles, and bicycles. Table 1 provides additional details regarding sociodemographic information.

The research data reflect that 339 (53.1%) of the studied school-age children were boys and 299 (46.9%) were girls; 67.4% of the studied children were in the primary grade level. This is because most rural areas have built village or township elementary schools, and rural school-age children are conveniently enrolled at the primary level, and at the secondary level, rural school-age children may go elsewhere for school choice.

The guardians of 63.7% of the researched children are over 40 years old, and more than half of them are grandparents of the researched children. The education level of the surveyed guardians is concentrated in primary school and below, and the highest education level of the family is generally concentrated in primary school and junior middle school. It can be seen that more young and middle-aged laborers prefer to go out to work, while grandparents stay in the village to take care of the children’s life and study. A total of 48.2% of the parents interviewed had an annual personal income of less than 10,000 yuan and a total household income of between 10,000 and 50,000 yuan. As the process of rural revitalization continues and the economy develops, nearly half of households own private cars, and the proportion of households with one electric bicycle is the highest, followed by private cars, bicycles, and motorcycles. Compared with motorcycles, rural residents prefer to choose lightweight, flexible, and affordable electric bicycles for daily travel.

#### 3.3.2. Variables Related to School Travel

In rural Sichuan, China, the main travel modes for school-age children to go to school are riding an electric bicycle and walking (account for 71.8%). In our preliminary survey, we found that the distance and time to go to school are the main factors affecting the travel mode choice of rural children, and parents and children have no idea about the cost of travel. As a result, this paper uses travel distance and travel time as variables related to school travel, shown in Table 2.

#### 3.3.3. School Travel Built Environment Perception and Relevant Variables

This study incorporates school travel built environment perceptions to explore their impacts on rural school-age children’s travel mode to school. The perception of school travel built environment refers to how rural school-age children feel about their current built environment. The perception of the built environment was scored on a five-point Likert Scale, which was used to calculate the satisfaction of school travel built environment. In the perception of school travel built environment, the scoring method for positive statements and attitude statements is 5 points for strongly agree, 4 points for agree, 3 points for general, 2 points for disagree, and 1 point for strongly disagree; the scoring method of negative statements is just the opposite: 1 for strongly agree, 2 for agree, 3 for general, 4 for disagree, and 5 for strongly disagree.

Rural school-age children’s perceptions of the school travel built environment shows whether the existing travel built environment meets some conditions, which contains 18 variables. The factor analysis of perceptions of the built environment was conducted using SPSS 24.0 with a Kaiser-Meyer-Olkin (KMO) value of 0.902. Factor rotation was performed using the maximum variance method. Finally, the 18 variables were grouped into four main compositions: school travel hardware facilities, road condition, accessibility factor, and safe environment (Table 3).

### 3.4. Multicollinearity of Variables

The multiple linear regression model is a commonly used method for prediction analysis. However, in practice, the problem of strong correlations between explanatory variables is called multicollinearity, which is due to flaws in experimental design or inherent linkages between variables [44,45,46]. In a given sample, if there is severe multicollinearity, the estimates calculated for the sample are biased [47] and may affect the model fit [48]. In this study, the variance inflation factor (VIF) was used to test the severity of multicollinearity. The VIF values of the independent variables selected are all less than 3 (Table 4), which is well below the VIF threshold of 10, indicating that there is no multicollinearity among the independent variables and that the next step of model fitting can be carried out.

In this study, before model fitting, the socio-demographic variables in household attributes and the number of transportation holdings variables were divided into two parts: parent attributes and household vehicle ownership. The personal attributes variables, parental attributes variables, household vehicle holdings variables, school travel built environment perception variables, and school travel variables were gradually incorporated into the model to explore the contribution of variables to the multinomial logit model. Firstly, the personal attribute variables were included in the model with a McFadden’s pseudo-R-squared of 0.092, indicating that the personal attribute variables contributed well to the model, and the likelihood ratio test is shown in Table 5.

Among the individual attributes, the age and gender variables were significantly less than 0.05, and the personal attribute variables had a significant effect on the choice of rural children’s mode of travel to school. Therefore, the personal attribute variables were retained and the parental attribute variables were brought into the model, whose McFadden’s pseudo-R-squared was 0.106, which was only 0.014 and more than the previous model of McFadden’s pseudo-R-squared, which shows that parental attributes did not contribute much to the model. Their likelihood ratio tests are shown in Table 6.

During the research process, the researchers found that families of students with better family conditions and private cars were more likely to choose private cars to commute to school. However, during the model-building process, the significance of all variables in parental attributes was greater than 0.05. This indicates that the family attribute variables in this study did not have a significant effect on the choice of school travel mode for rural children, so the family factor was excluded from the final multinomial logit model. Therefore, in the final model, the degree of influence of parental attributes on children’s choice of mode of commuting to school was not considered.

After excluding the parental attribute variable, the household vehicle ownership variable was put into the model with a McFadden pseudo-R-squared of 0.133, which is an increase of 0.041 from the first McFadden model, and the household vehicle ownership variable contributed well to the model. Its likelihood ratio test is shown in Table 7.

The results of the likelihood ratio test of the independent variables show that the *p*-values of significance for children’s gender and age are less than 0.05, and the *p*-value of significance for bicycle ownership in the household vehicle ownership variable is less than 0.05. This indicates that this variable affects the choice of rural children’s mode of travel to school, so the number of means of transportation variable is retained.

The McFadden pseudo-R-squared for children’s perceptions of the school travel built environment was 0.223, an increase of 0.09 over the McFadden pseudo-R-squared for the previous model while retaining the personal attributes variables and the household vehicle ownership variable. Children’s perceptions of the built environment variable contributed better to the model. Their likelihood ratio tests are shown in Table 8.

In addition to the personal attribute variables and household vehicle ownership variable, the significance *p*-values of the three variables of the perception of the built environment: hardware facilities, road conditions, and safe environment were less than 0.05. This indicates that the perception of the built environment affects rural children’s choice of school travel mode. Finally, the children’s distance to school travel variable was brought into the model, and this model of McFadden’s (McFadden’s) pseudo-R-squared grew to 0.383, indicating that the distance to school variable contributed well to the model, and its likelihood ratio test is shown in Table 9.

## 4. Results and Discussion

In the results of the likelihood ratio test for the independent variables of this multinomial logit model, the personal attribute variable, the household vehicle ownership variable, the perception of the school travel built environment variable, and the distance to school variable had good significance. The results of model fit parameter estimation are shown in Table 10.

### 4.1. Personal Attributes and Household Vehicle Ownership

Among children’s personal attributes, age and gender have a significant impact on children’s choice of school travel mode, and their significance is lower than 0.1, as shown in Table 10. Compared with walking, the probability of children choosing private cars (−0.139), electric bicycles (−0.252), and motorcycles (−0.376) to travel are all negatively correlated with age, and the probability of choosing bicycles (0.288) and buses (0.162) is positively correlated with age. This indicates that the older children are, the less likely they are to choose private cars or electric bicycles for school travel, and the more likely to choose independent ways for school travel, such as bicycles, buses, or walking. This is consistent with existing studies, where age is considered as a key factor in either independent school travel or parental pick-up and drop-off patterns [49,50], because younger children are more likely to be escorted to school [51,52] potentially due to personal and safety considerations [53,54]. In contrast, older students are more eager to travel to school independently with more cognitive ability. In general, children become more independent as they grow older [50].

Many children prefer to take public transport for social interaction than to walk to/from school alone [55]. Experience from other countries shows that as people become more affluent, private car ownership increases, thus contributing to the growth of carbon emissions. Promoting the use of public transport is one of the key strategies to reduce the traffic emissions [56]. Although buses do not promote physical activity as much as walking, bus rides are beneficial in fostering children’s independence and developing their habits of using public transport, which has a positive long-term impact on sustainability. 

Compared with girls, boys prefer to travel by bike (2.079). This fact is consistent with other surveys [57] where girls prefer to be escorted by a car, while boys prefer to walk or bicycle to school [58]. Gender also has an impact on the independence of school travel [51,52] because parents may be more worried about the safety of girls. In rural areas, parents are particularly concerned about the safety of girls, especially younger girls who are rarely allowed to go to school without parental supervision.

Among household vehicle ownership, only household motorcycle ownership and bicycle ownership have a significant impact on rural children’s choice of motorcycles and bicycles to go to school. Household bicycle ownership (1.328) has a positive impact on rural children’s choice of bicycle traveling to school, while household motorcycle ownership (−2.396) has a significant negative correlation with rural children’s choice of traveling to school. Household motorcycle ownership (−2.396) has a significant positive correlation with rural children’s choice of motorcycle general learning, while electric bicycle ownership has a significant negative impact. 

From the literature, we see that Lin et al. [59] took the number of bus lines, car ownership, motorcycle ownership, and bicycle ownership as the mode selection variables and found that increasing the bus route or service frequency during children’s going to/from school is conducive to children’s independent bus use. Both McMillan [60] and McDonald [17] concluded that household income is positively correlated with children’s car use. However, these relationships are not significant in this study. It can be explained that in recent years, the rural economy has been developing and the economic conditions of rural residents have gradually improved, which has led to a continuous increase in the number of cars owned by rural families. However, the convenience, money-saving, and environmental advantages of electric bicycles have led almost every household in rural areas to purchase them. Unlike the daily trips of rural residents, children have a more fixed time and place to go to school. Within a certain distance, rural residents prefer to use convenient and cost-effective electric bicycles to pick up their children to and from school. Therefore, a non-significant effect of household car ownership on children’s choice of school passages was found in this study.

### 4.2. Built Environment Perception

Regarding the perception of the built environment of travel to school, when rural school-age children perceive better conditions of hardware facilities for access to school, the highest probability of choosing the electric bicycle (0.346) for access to school is shown, followed by walking, private car (−0.410), and bus (−0.611). When rural school-age children perceive that the road conditions to school are better, the road to school has low speed and is not congested in front of the school, they are most likely to choose the electric bicycle (0.450) to go to school, followed by walking, and private car (−0.376). It can be explained that in rural areas, the electric bicycle is recognized as the most convenient and cost-saving means of transportation. Among the roads with good road conditions and facilities, they prefer the electric bicycle because it is time and labor saving. When the distance from the residence to the school is short and the number of intersections is small, and rural school-age children think that the school is convenient and accessible, and they are most willing to choose bicycles (0.107) and motorcycles (0.051), followed by walking, private cars (−0.674), and buses (−0.699). If the environment at the school gate is safe, children are most likely to use bicycles (0.653) to travel to school, followed by walking, and finally private cars (−0.520). The study found that a safe, convenient, and well-equipped school travel built environment can promote children to choose convenient and fast school travel modes.

### 4.3. School Travel Distance

The distance from home to school is an important factor affecting the choice of children’s school travel modes. Many scholars have also demonstrated in their research that distance is an important factor affecting children’s school travel modes. The distance from home to school is a significant barrier to walking or cycling to school [61]. Consistent with the findings of previous studies, as shown in Table 10, distance has a significant positive correlation with the impact of bicycles (2.051), motorcycles (2.023), electric bicycles (2.657), buses (2.890), and private cars (2.927). In this study, the effect of travel distance on school commuting by motorcycle (2.023) was smaller than that of electric bicycle (2.657). This was due to the low number of rural school-age children who used motorcycles as their daily means of transportation to and from school in this study, and the parents who use motorcycles to pick up and drop off their children from school are older. With shorter distances and better accessibility to home and school, they are more willing to choose to pick up their children by motorcycle.

The greater the distance, the more children prefer to take a private car through school, which is efficient and fast while taking the bus requires a corresponding bus stop and a suitable bus route. In contrast, it is more convenient and faster to take a private car, which is consistent with the findings on the effect of distance on travel mode choice among rural residents [62].

## 5. Conclusions

The rural school-age children’s travel to and from school behavior is an important part of rural transportation, and their travel has also changed the travel structure of some families. However, the travel experience of school-age children is not as good as that of adults, and children have a certain degree of dependence. There is a certain gap between the existing planning and construction of facilities in rural areas and cities, and children’s travel factors are not sufficiently considered. This research collected research data through household surveys, questionnaires, and field measurement methods, established a multinomial logit (MNL) model, analyzed the impact of factors on students’ choice of travel mode, and considered the impact of rural children’s perception on the school travel built environment.

Among the personal attribute variables, the choice of bicycle and bus travel by rural children is significantly and positively correlated with age, while the choice of electric bicycle and private car to go to school is significantly and negatively correlated with gender; bicycle ownership is significantly and positively associated with children’s choice of bicycle traveling behavior, and motorcycle ownership is significantly and negatively associated with children’s choice of bicycle traveling. 

In the perception of the school travel built environment, children who go to school on a path with good access to school facilities are most likely to choose to take an electric bicycle, followed by walking. When the road conditions are better and traffic speed is low without congestion, they are most willing to choose the electric bicycle. When children feel that it is convenient to go to school, they are more willing to choose bicycles and motorcycles. When children perceive that the public security environment at the school gate is good, they are willing to go to and from school by bike or on foot.

The distance between children’s residence and school is significantly positively correlated with the travel of bicycles, motorcycles, electric bicycles, buses, and private cars, and private cars have the most significant impact.

Through the investigation and research, it was found that the frequency of choosing bicycles and motorcycles in the school travel mode of rural school-age children is very low. At present, the better-developed rural areas have eliminated bicycle travel. With the rise in oil prices, the probability of rural residents using motorcycles to pick up children to and from school has also decreased. In China’s statistical yearbook, the number of bicycles and motorcycles owned by farmers has continued to decline in recent years, replaced by more cost-effective and labor-saving electric bicycles. Rural school-age children use more private cars and buses for long-distance school travel. Good accessibility promotes rural school-age children to walk to school.

China has entered a stage of rapid development of new countryside and urbanization. The advancement of urbanization has transformed rural areas from traditional scattered living to centralized living, and the changing rural built environment is changing the way rural children travel to and from school. In recent years, scholars have gradually increased research on transportation planning and residents’ travel, but few studies have evaluated the development of rural transportation construction from the perspective of rural children. Therefore, this study reflects the needs of rural children to travel to school through the change in rural children’s travel mode. The results of this study are intended to provide reference opinions for rural construction and urban and rural planning in Chengdu, Sichuan Province, and are pertinent and practical. To realize the optimization of rural traffic construction, it is of great significance for the construction of rural road networks and the planning and development of rural traffic to put forward suggestions on the deficiencies in the construction of rural roads and infrastructure. 

To sum, this study used the rural areas in Chengdu as the research object, which enriches the research on rural areas and provides a reference and theoretical basis for the future rural research from the perspective of children, especially in Southwest China. Moreover, reasonable suggestions are put forward for the construction of rural road infrastructure and environmental construction in Sichuan. The proposed suggestions are more desirable based on the actual environment of Sichuan Province. 

### 5.1. Policy Implications

Based on the characteristics of the influences of various variables on school travel mode choice, this study provides enlightenment for the new rural road construction:

1. While road construction meets the needs of more rural residents to drive and ride electric bicycles, it should improve the safety of school-age children in rural areas;
2. Consider adding sidewalks/bicycle paths to meet the needs of rural school-age children to go to school by bike/on foot, protect children’s safety, and promote children to go to school positively;
3. The low use of public transport among rural children is related to the poor construction of public transportation facilities in new rural areas, as well as the accessibility and frequency of public transport. Attention to the construction and maintenance of public transport infrastructure in the new countryside is needed in the future.

### 5.2. Limitations

Several limitations of this study are listed below.

1. The scope of the study is limited. The selected villages are all from Chengdu city, and the sample villages are selected by distance from the center of Chengdu city to the periphery. Although these locations are somewhat representative, they cannot fully represent other rural areas in the country, so there are some limitations in the regional distribution of this research;
2. This study did not find a significant correlation between some family factors such as parental age, education, and annual household income and children’s choice of school passages, which is not consistent with other studies and this deems further investigation;
3. The scope of the study was limited and the sample size obtained was limited. There are certain limitations in the regional distribution of the sample villages in the study of rural school children’s travel mode choice;
4. The model used can be also compared with other potential models to evaluate the results more thoroughly.

## Figures and Tables

**Table 1:** Sociodemographic variables.

Personal Attributes		Frequency	Personal Attributes		Frequency
Gender	Male	53.1%	Only child	Yes	62.9%
	Female	46.9%		No	37.1%
Age	6–12 years old	69.0%	Grade	1–6	67.4%
	13–15 years old	22.0%		7–9	22.7%
	15–18 years old	8.9%		10–12	9.9%
Gender	Male	36.1%	Relationship with the children	Father	14.6%
	Female	63.9%		Mother	29.0%
Age	Under 30 years old	5.0%		Grandfather	24.3%
	31–40 years old	31.5%		Grandmother	31.8%
	41–50 years old	11.5%		Other	0.3%
	51–60 years old	35.5%	Annual personal income	Less than CNY 10,000	48.2%
	60 years old or older	16.7%		CNY 10,000–50,000	39.7%
Education level	Primary school and below	51.3%		CNY 50,000–100,000	11.1%
	Junior high school	24.3%		CNY 100,000–150,000	1.6%
	High school or junior college	20.1%	Driver’s License	Yes	31.0%
	College degree	2.4%		No	69.0%
	Bachelor’s degree	1.9%	Can ride a motorcycle/electric bicycle	Yes	89.7%
	Master’s degree and above	0.2%		No	10.3%
Family members	3 and under	8.8%	Total household income	CNY 10,000–50,000	30.1%
	4	19.0%		CNY 50,000–100,000	46.0%
	5	41.1%		CNY 100,000–150,000	18.4%
	6 and above	31.1%		CNY 150,000–200,000	5.2%
				More than CNY 200,000	0.3%
Number of cars	0	52.2%	Number of motorcycles	0	81.2%
	1	45.9%		1	18.5%
	2	1.7%		2	0.3%
	3 or more	0.2%		3 or more	0
Number of electric bicycles	0	6.0%	Number of bicycles	0	74.7%
	1	83.7%		1	24.8%
	2	8.9%		2	0.3%
	3 or more	1.4%		3 or more	0.4%

**Table 2:** School travel variables.

Personal Attributes		Frequency	Personal Attributes		Frequency
Distance	<500 m	12.2%	Time	<10 min	38.1%
	500 m–1 km	8.6%		11–20 min	47.3%
	1–2.5 km	43.6%		21–30 min	7.5%
	>2.5 km	35.6%		>30 min	7.1%

**Table 3:** Component matrix of school travel built environment perception.

Variable	Composition			
	Hardware Facilities	Road Conditions	Accessibility	Safe Environment
Wide pathways to school	0.837	−0.042	0.062	0.038
Road leveling on the way to school	0.808	−0.081	0.034	0.056
No damage to the pathway to school	0.799	−0.130	0.036	−0.002
No obstacles on the way to school	0.760	−0.092	0.138	0.111
Speed bumps around the school set up reasonably	0.792	−0.003	0.045	−0.141
Wide view around the school	0.830	0.028	0.025	0.011
Reasonable location of school entrances and exits	0.737	−0.063	0.256	0.019
Speed limits, parking, and other signs set up reasonably	0.714	−0.053	0.268	0.113
Plenty of greenery on the way to school	0.709	−0.087	0.181	0.070
Fun on the way to school, with play and fitness facilities	0.631	−0.011	0.185	0.032
Good lighting conditions on the way to school	0.616	0.107	0.418	0.220
Fast motor vehicle speed on the way	0.112	0.879	−0.180	−0.046
Motor vehicle speed makes travel dangerous	−0.136	0.879	−0.038	0.032
Congestion in front of the school during the school day	−0.233	0.536	0.166	0.329
Reasonable distance from home to school	0.098	−0.070	0.730	−0.036
Low number of intersections on the way to school	0.352	−0.098	0.549	−0.312
Mobile vendors at the school gate will not affect you	0.097	0.061	−0.184	0.810
Good security control and a safe security environment around the school	0.404	0.087	0.443	0.498
Summary Statistics				
Characteristic value	7.115	2.003	1.104	1.057
Percentage variance	39.530	11.130	6.133	5.872
Cumulative variance percentage	39.530	50.660	56.793	62.665

Note: extraction method: principal component analysis; rotation method: varimax with Kaiser normalization; rotation converged in six iterations.

**Table 4:** Multicollinearity test.

Explanatory Variables	VIF	Explanatory Variables	VIF
Personal attributes		Family attributes	
Gender	1.038	Parental gender	1.136
Age	1.181	Parental age	1.589
Only child or not	1.154	Total number of family members	1.169
School travel variables		Annual household income	1.351
Distance to travel to school	1.324	Whether you have a driver’s license	2.004
School travel built environment perception		Whether you can ride a motorcycle (electric bicycle)	1.117
Hardware Facilities	1.113	Number of motorcycles owned by households	1.085
Road conditions	1.035	Number of electric bicycles owned by households	1.058
Accessibility	1.126	Number of households owning private cars and vehicles	1.625
Safe environment	1.095	Number of bicycles owned by households	1.118

**Table 5:** Likelihood ratio test for independent variables.

Variables	Model Fitting Conditions	Likelihood Ratio Test		
	Simplified Model 2 Log Likelihood	Bangla	Degree of Freedom	Significant
Constant	500.464	0.000	0	
Gender	623.823	123.259	5	0.000
Age	516.188	15.724	5	0.008
Only child or not	504.399	3.935	5	0.559

**Table 6:** Likelihood ratio test for independent variables.

Variables	Model Fitting Conditions	Likelihood Ratio Test		
	Simplified Model 2 Log Likelihood	Bangla	Degree of Freedom	Significant
Constant	1430.256	0.000	0	
Gender	1552.147	121.891	5	0.000
Age	1445.859	12.602	5	0.008
Only child or not	1436.127	5.871	5	0.319
Total number of family members	1434.921	4.664	5	0.458
Parental gender	1434.936	4.679	5	0.456
Parental age	1432.066	1.810	5	0.875
Whether you have a driver’s license	1432.347	2.090	5	0.836
Whether you can ride a motorcycle (electric bicycle)	1439.118	8.861	5	0.115
Annual household income	1432.408	2.152	5	0.828

**Table 7:** Likelihood ratio test for independent variables.

Variables	Model Fitting Conditions	Likelihood Ratio Test		
	Simplified Model 2 Log Likelihood	Bangla	Degree of Freedom	Significant
Constant	1053.640	0.000	0	
Gender	1172.632	118.992	5	0.000
Age	1068.634	14.994	5	0.010
Only child or not	1058.138	4.498	5	0.480
Number of motorcycles	1073.027	19.387	5	0.002
electric bicycles	1063.671	10.030	5	0.074
Number of private cars	1056.142	1.502	5	0.776
Number of bicycles	1090.437	36.797	5	0.000

**Table 8:** Likelihood ratio test for independent variables.

Variables	Model Fitting Conditions	Likelihood Ratio Test		
	Simplified Model 2 Log Likelihood	Bangla	Degree of Freedom	Significant
Constant	1243.130	0.000	0	
Gender	1344.208	101.078	5	0.000
Age	1255.050	11.920	5	0.036
Only child or not	1245.685	2.554	5	0.768
Number of motorcycles	1260.632	17.502	5	0.004
electric bicycles	1252.802	9.672	5	0.085
Number of private cars	1245.703	2.573	5	0.765
Number of bicycles	1269.535	26.405	5	0.000
Hardware Facilities	1296.684	53.554	5	0.000
Road conditions	1286.569	43.439	5	0.000
Accessibility	1250.390	7.259	5	0.202
Safe environment	1279.861	36.731	5	0.000

**Table 9:** Likelihood ratio test for independent variables.

Variables	Model Fitting Conditions	Likelihood Ratio Test		
	Simplified Model 2 Log Likelihood	Bangla	Degree of Freedom	Significant
Constant	988.596	0.000	0	
Gender	1072.190	83.594	5	0.000
Age	996.815	8.218	5	0.145
Only child or not	992.629	4.033	5	0.545
Number of motorcycles	1008.860	20.263	5	0.001
Electric bicycles	1000.530	11.934	5	0.036
Number of private cars	989.659	1.062	5	0.957
Number of bicycles	1006.843	18.246	5	0.003
Hardware Facilities	1028.974	40.378	5	0.000
Road conditions	1025.823	37.226	5	0.000
Accessibility	1014.399	25.802	5	0.000
Safe environment	1014.174	25.578	5	0.000
Distance to travel to school	1244.516	255.920	5	0.000

**Table 10:** MNL model parameter estimation.

	Bicycle		Bus		Car		Electric Bicycle		Motorcycle	
	B	*p*	B	*p*	B	*p*	B	*p*	B	*p*
Intercept	−7.829	0.000	−6.660	0.000	−2.579	0.013	0.853	0.287	1.333	0.538
Sociodemographic variables										
Age	0.288	0.029	0.162	0.043	−0.139	0.050	−0.252	0.000	−0.376	0.020
Male (Female = ref.)	2.079	0.042	0.172	0.703	−0.203	0.612	−0.043	0.892	−0.248	0.772
Only child (not an only child = ref.)	−0.246	0.682	0.023	0.959	0.335	0.413	0.187	0.563	−1.181	0.204
Household vehicle ownership										
Number of motorcycles	−2.396	0.036	−0.569	0.314	−0.597	0.232	−0.567	0.149	2.333	0.012
Number of electric bicycles	−1.357	0.126	−0.384	0.440	−0.631	0.189	−0.066	0.861	−3.080	0.007
Number of private cars	−0.522	0.378	0.047	0.912	−0.028	0.941	0.009	0.976	0.151	0.851
Number of bicycles	1.328	0.008	−0.258	0.598	0.121	0.774	−0.123	0.721	−0.490	0.555
School travel built environment perception										
Hardware Facilities	−0.079	0.831	−0.611	0.009	−0.410	0.048	0.346	0.025	−0.553	0.185
Road conditions	−0.193	0.524	0.167	0.466	−0.376	0.082	0.450	0.006	0.157	0.727
Accessibility	0.107	0.049	−0.699	0.003	−0.674	0.003	−0.151	0.405	0.051	0.047
Safe environment	0.653	0.037	−0.233	0.305	−0.520	0.012	0.081	0.622	−0.680	0.146
School travel-related variables										
Distance to travel to school	2.051	0.000	2.890	0.000	2.927	0.000	2.657	0.000	2.023	0.000

## Data Availability

The data used to support the findings of this study are available from the corresponding author upon request.
